# Off-Label Baclofen Prescribing Practices among French Alcohol Specialists: Results of a National Online Survey

**DOI:** 10.1371/journal.pone.0098062

**Published:** 2014-06-02

**Authors:** Benjamin Rolland, François Paille, Benoit Fleury, Olivier Cottencin, Amine Benyamina, Henri-Jean Aubin

**Affiliations:** 1 Société Française d’Alcoologie, Issy-les-Moulineaux, France; 2 Service d’Addictologie, CHU Lille, Univ Lille Nord de France, Lille, France; 3 Service d’Addictologie, CHU Nancy, Université, Univ Nancy, Nancy, France; 4 Service d'Hépatogastroentérologie et d'Alcoologie, CHU Bordeaux, Bordeaux, France; 5 CERTA L’Albatros, Hôpital Paul Brousse, AP-HP, INSERM U 669, Villejuif, France; University of Granada, Spain

## Abstract

**Objective:**

To evaluate, among alcohol specialists belonging to the *Société Française d’Alcoologie* (SFA), i.e., the French Alcohol Society, the proportion of physicians who prescribed off-label baclofen for alcohol use disorders (AUDs). The secondary objective was to depict the features of individual prescribing and monitoring practices.

**Methods:**

On-line survey among 484 French alcohol specialists. Physicians were asked whether they prescribed baclofen for AUDs. If they did not, the reasons for this choice were investigated. If they did, the features of the physician’s prescribing practice were explored, including the number of patients treated, the mean and maximum doses, the monitoring precautions and the pharmacovigilance reporting. Participants were also asked about their empirical findings on HDB’s efficacy and safety.

**Results:**

In total, 302 physicians (response rate of 62.4%) participated in the survey. Data from 296 participants were analysed, representing 59.4% of all active prescribing physicians belonging to the SFA. HDB use was declared by 74.6% of participants (mean dose 109.5±43.6 mg/d; maximum dose 188±93.3 mg/d). However, 79.2% of prescribers had treated less than 30 patients, and 67.8% used HDB as a second-line medication. Although HDB was perceived as more efficacious than approved drugs by 54.3% of prescribers, it was also declared less safe by 62.8%. Nonetheless, 79.7% of prescribers had never filed any pharmacovigilance report. Non-prescribers (25.6%) were primarily deterred by the current lack of scientific data and official regulation.

**Conclusion:**

A majority of French alcohol specialists reported using HDB, although often on a limited number of their patients. HDB was considered efficacious but also potentially hazardous. Despite this, physicians reported minimal safety data to the health security system. While French health authorities are planning to draft a specific regulatory measure for framing off-label HDB prescribing practices, the sustained education of prescribers on spontaneous pharmacovigilance reporting should be enhanced.

## Introduction

Baclofen is a gamma amino-butyric acid type B (GABA-B) receptor agonist. It has been a long-standing treatment for spasticity [1] and was approved in France in 1974. Over the last decade, baclofen has emerged as a promising medication for addictive disorders, particularly for alcohol use disorders (AUDs) [2], [3]. To date, most evidence-based data on baclofen for AUDs have been gathered from studies experimenting with a dose of 30 mg/d in patients meeting the criteria for alcohol dependence [4]–[6]. At this dose, the efficacy results of baclofen are contradictory, as two studies showed a significant benefit versus placebo [4], [5], whereas another found no difference [6].

In parallel with these research investigations, several case reports [7], [8], case series [9], and open-label cohort studies [10], [11] have highlighted that high-dose baclofen (HDB), i.e., more than 80 mg/d and sometimes up to 400 mg/d, has also been used empirically in daily clinical practice. These off-label prescribing practices of HDB emerged and developed primarily in France after the publication of an auto-case report [7], followed by an autobiographic general public book published in 2008 [12], in which a French cardiologist maintained that the self-prescription of HDB completely and durably suppressed his dependence on alcohol. Heavy media coverage was given to the story of this physician [13], which led to a national demand for baclofen within the general public and among numerous practitioners. As a result, baclofen’s sales increased by 20% between 2008 and 2010 [14].

The unevidenced and unregulated aspect of such prescribing practices has deeply dismayed the French medical community. On one hand, no solid efficacy data were available on HDB, and in terms of public health, the safety profile of a sedative drug such as HDB was completely unknown, particularly in patients with sometimes-heavy alcohol drinking patterns and frequent comorbidities and comedications. Recent reports have suggested that baclofen use is associated with safety issues in patients with AUDs [15]–[16]. On the other hand, some of the patients who were treated with HDB have gathered into patient associations that vigorously lobby to obtain official approval of HDB for AUDs. In practice, the attitudes among physicians have been quite split. Some chose to join the pro-baclofen associations, sometimes lobbying in the media for the more widespread use of HDB to treat AUDs. Conversely, other physicians have chosen not to prescribe baclofen until an official approval has been delivered for it. Last, in the middle, some physicians have opted for more pragmatic approaches, prescribing baclofen only at low doses or only when approved treatments have failed. Some teams have even proposed the implementation of specific systems for cautiously monitoring patients under HDB and these teams systematically report adverse events to the pharmacovigilance system [17].

This particularly uncommon situation has induced some equally uncommon public health issues. The lack of any official regulation or guidelines about how to prescribe and monitor HDB has led to the development of a multitude of home-made prescribing practices. Each prescriber has had to decide almost everything by themselves: the types of patients to whom baclofen should be prescribed, the maximum reachable dose, the best dose escalation pattern, the most appropriate treatment duration, and the best way to monitor patients. Indeed, such individual prescribing practices have developed in a complete fog, and feedback based on the public health data has remained cursory. In 2012, a national pharmacovigilance report noted that although HDB appeared to be associated with frequent and sometimes harmful adverse events, there was a dramatic under-reporting of pharmacovigilance data [16]. Moreover, although the law requires the reporting of ‘hors-AMM’, i.e., ‘off-label’, use on HDB prescriptions, it is likely that many physicians do not adhere to this requirement because they simply ignore the law or because such a mention implies that the treatment would not be reimbursed to the patient.

Faced with this situation, the *Agence Nationale de Sécurité du Médicament et de produits de santé* (ANSM), the French medicines and healthcare products regulatory agency, has been in a tight manoeuvring situation. On one hand, they could not forbid physicians to prescribe HDB, and they had to face the extreme pressure of the pro-baclofen associations. On the other hand, they had to remain alert to the safety concerns that followed from this off-label prescribing practice. In June 2013, the ANSM announced that they intended to publish ‘Temporary Recommendations for Use’ (TRU) for baclofen. The TRU are a new measure for regulating specific off-label drug uses in France [18]. A TRU on HDB should allow the homogenisation and rationalisation of practices, avoiding the most harmful prescribing patterns, and will most likely restrict the use of HDB to specific situations or specific types of AUD patients. However, there are two issues with this TRU project. First, the scientific literature will be of almost no help because the current data available on HDB are both limited and considered to have a low quality of evidence. Moreover, the empirical prescribing practices have been poorly described, both in their number and their diversity.

In expectation of the TRU and to provide a clearer overview of the current off-label prescribing practices of baclofen among French alcohol specialists, the *Société Française d’Alcoologie* (SFA), i.e., the French Society for Alcohol, conducted an online survey amongst their members. Although not all of the SFA physician members are prescribing physicians, the SFA physician members all take part in professional activities closely related to research, prevention, or care regarding AUDs. Among all clinician members of the SFA, treating patients with AUDs constitutes a large part of their daily professional activity.

## Materials and Methods

### Study Design and Participants

This survey investigated the off-label prescription practices for HDB in the treatment of AUDs by physician members of the SFA. Among the 622 physician members of the SFA, the 484 who had provided a valid email address were contacted via email and asked to complete an anonymous online survey. The survey was opened online on July 1, 2013, and closed on July 17, 2013. After an initial email invitation was sent on July 1, 2013, non-responders received two consecutive reminder emails on July 8^th^ and July 11^th^. Each participant could complete the questionnaire only once, as the personal link for reaching the questionnaire was deactivated after completion of the survey.

### Data Collection

All of the participants were asked about their age and gender. They were then asked the question ‘*Do you prescribe baclofen for alcohol use disorders?*’ Describing the response pattern to this question was defined as the main objective of the survey. Depending on the participants’ answers to this first question, a specific question tree followed. The tree is shown in **Table 1**.

**Table 1:** Survey questions and answers.

Physicians who never prescribe baclofen in AUDs; n = 76 (25.5%)	
**1) Why do not you prescribe baclofen** **(multiple choice)?**	1) There is a lack of scientific evidence (81.3%); 2) There are risks in prescribing off-label (41.2%); 3) I do not prescribe anticraving drugs (6,3%); 4) I lack personal training for using baclofen (25.4%); 5) After trying, I think baclofen is ineffective (7.9%); 6) After Trying, I think baclofen induced too many adverse drug reactions (6.3%)
**2) Would you consider prescribing baclofen if** **TRU are published?**	1) Yes (29.5%); 2) No (21.3%); 3) Do not know (49.2%);
**Physicians who prescribe baclofen in AUDs; n = 222 (74.5%)**	
**A) For how many of your patients have you** **prescribed baclofen for treating AUDs?**	1) <10 (36.5%); 2) 11–30 (42.7%); 3) 31–60 (11.8%); 4) >60 (9%)
**B) Before prescribing baclofen, have you previously** **tried an approved treatment?**	1) Never (16.3%); 2) Sometimes (15.9%); 3) Often (28.3%); 4) Always (39.5%)
**C) What is the main treatment goal when you** **prescribe baclofen (multiple choices)?**	1) Reduction in alcohol drinking (21.5%); 2) Abstinence maintenance (23%); 3) Both (55.5%)
**D) In which situation do you prefer starting baclofen?**	1) Mainly in already-abstinent patients (24.9%); 2) Mainly in non-abstinent patients (32.2%); 3) Both equally (42.9%)
**E) What is the scheme of dose escalation you usually** **follow in your prescriptions?**	1) <10 mg/w (4.3%); 2) 10 mg/w (56%); 3) 20 mg/w (14.5%); 4) 30 mg/w (18.8%); 5) 40 mg/w (0%); 6) 50 mg/w (0.5%); 7) 60 mg/w (3.4%); 8) 70 mg/w (0%); 9)>70 mg/w (2.5%)
**F) Have you set a maximum dose of** **baclofen you usually never go over (mg/d)?**	1) Yes (61.2%) 2) No (38.8%)
**G) What is the maximum dose of baclofen that you** **have ever prescribed (mg/d)?**	188±93.3 mg/d
**H) What is the approximate mean dose of baclofen** **that you prescribe (mg/d)?**	109.5±43.6 mg/d
**I) What is the mean period during which you use** **baclofen at maximum dose?**	1) <3 months (21.1%); 2) 3–6 months (37.3%); 3) 6–12 months (28.3%); 4) >12 months (13.3%)
**J) After a period at maximum dose, do you usually** **attempt to reduce baclofen at a lower dose to reduce ADRs?**	1) Never (3.7%); 2) Sometimes (27%); 3) Often (49.7%); 4) Always (19.6%)
**K) How do you divide doses across the day?**	1) Divided in 3 equivalent doses at each meal (57.4%); 2) According to patient’s craving or tolerance (39.6%) 3) >3 doses per day (2.4%); 4) Other (0.6%)
**L) How often do you associate baclofen with an** **approved treatment (acamprosate, naltrexone, or disulfiram)?**	1) Never (53.4%); 2) Sometimes (38.5%); 3) Often (7.5%); 4) Always (0.6%)
**M) What is your personal opinion on** **baclofen’s efficacy, by comparison with approved treatments?**	1) Much more efficacious (16.3%); 2) More efficacious (38%); 3) Equally efficacious (36.7%); 4) Less efficacious (8.4%); 5) Much less efficacious (0.6%)
**N) What is your personal opinion on baclofen’s** **safety, by comparison with approved treatments?**	1) Much safer (1.2%); 2) safer (5.8%); 3) Equally safe (30.4%); 4) Less safe (50.3%); 5) Much less safe (12.3%)
**O) What proportion of your patients was** **significantly and durably improved with baclofen (%)?**	1) 0% (4.2%); 2) 10% (10.3%); 3) 20% (13.3%); 4) 30% (15.2%); 5) 40% (9.7%); 6) 50% (24.2%); 7) 60% (10.3%); 8) 70% (8.5%); 9) 80% (4.3%); 10) 90% (0%); 11) 100% (0%)
**P) What proportion of your patients had to stop** **baclofen because of ADRs (%)?**	1) 0% (5.2%); 2) 10% (23.3%); 3) 20% (26.2%); 4) 30% (16.9%); 5) 40% (9.3%); 6) 50% (7%); 7) 60% (4.1%); 8) 70% (2.3%); 9) 80%(2.3%); 10) 90%(2.3%); 11) 100% (1.1%)
**Q) How many ADRs have you reported to** **pharmacovigilance units or to the ANSM?**	1) 0 (79.7%); 2) 1–4 (13.7%); 3) 5–29 (5.9%); 4) >30 (0.7%)
**R) How often do you write ‘** ***hors-AMM*** **’ on your prescription?**	1) Never (48.6%); 2) Sometimes (12.1%); 3) Often (9.2%); 4) Always (30.1%)
**S) How often do you write ‘** ***NR*** **’ on your prescription?**	1) Never (69.5%); 2) Sometimes (9.2%); 3) Often (6.3%); 4) Always (15%)

After defining their age and gender, participants had to precise whether or not they prescribe baclofen for AUDs in their daily practice.

Physicians who indicated that they do not prescribe baclofen had to complete only two additional questions, whereas physicians who declared prescribing baclofen for AUDs were asked to complete 17 additional questions. Response patterns to each question are noted in brackets.

AUDs = Alcohol Use Disorders.

TRU = ‘Temporary Recommendations for Use’. TRU is a new official measure for regulating the off-label prescribing practices in France. Baclofen will be the first drug to which TRU will be applied.

ADRs = Adverse Drug Reactions.

Hors-AMM = ‘Hors Autorisation de Mise sur le Marché’, i.e., ‘Out of Approval’. This mention is theoretically compulsory on any off-label prescription, but it involves that the treatment cannot be reimbursed to the patient.

NR = ‘Non remboursable, i.e., ‘Non-reimbursable’. This was the former compulsory mention to add on the prescription. It has no legal value anymore, since the last law that redefined in 2011 the conditions for prescribing off-label in France.

### Statistical Analyses

Descriptive statistics were performed on the whole sample (all responders) and on the two different groups (baclofen prescribers and non-prescribers) for the questions specifically fitted for each group. Additional descriptive analyses were also performed on non-responders of the survey and on the group of physicians for whom no email address was available and who were therefore not asked to participate in the survey.

Univariate analyses were performed to compare the features of responders, using the Wilcoxon signed-rank test for continuous variables and either the Chi-square or Fisher exact test for categorical variables. A weighted mean of percentages was calculated for questions O and P (**Table 1**). Quantitative variables (questions G and H) were also divided in rank-ordered deciles to calculate descriptive statistics and conduct multiple subgroup analyses.

Comparisons within multiple subgroups, e.g., within answer patterns to ordered-category questions or within dose-decile subgroups, were performed using Kruskal-Wallis H-tests for continuous variables and Chi-square tests for categorical variables. Response associations between answers to two ordered-category questions or between answers to an ordered-category question and a quantitative variable were determined using Spearman’s rank correlation coefficient (ρ). Analyses were performed using *XLSTAT2013* software (Addinsoft, Paris, http://www.xlstat.com). The significance level was set at 5% for all tests.

### Ethics Statement

The survey design was not previously approved by an ethics committee, as French law does not impose this procedure for a survey of physicians. However, the data were de-identified before being analysed, and the details of the data analysis were declared to the *Commission National Informatique et Liberté*s (CNIL). In accordance with the CNIL requirements, the participants of the survey were informed that the data they have provided can be used for publication, unless they expressly oppose to it.

## Results

**Table 1** lists the complete question tree of the survey and describes the response patterns of these questions.

Among 622 physician members affiliated with the SFA, 484 were invited by email to participate in the survey. Responses were received from 302 (62.4%) of the 484 invited SFA members.

The distribution of retired, active prescribing, and active non-prescribing physicians among the groups of uninvited, invited but non-responding, and invited and responding SFA members is shown in **Table 2**. Non-prescribing SFA physician members were typically occupational physicians or physicians who exclusively worked in public health or in a research institute. Among the 302 participants, the responses of six subjects were excluded from the analysis because they were non-prescribing physicians (n = 4) or because they were retired (n = 2). Therefore, among the 498 active prescribing physicians who belonged to the SFA, 296 (59.4%) participated in the survey. Moreover, the participation rate was 69.4% among the invited prescribers.

**Table 2:** Repartition of active prescribing, active non-prescribing, and retired physicians within the groups of SFA members who were not invited to participate in the survey, who were invited but did not respond, and who were invited and participated.

	Uninvited physicians (no valid email)	Non-responders	Responders	TOTAL
**Active prescribers**	72	130	296	**498**
**Active non-prescribing physicians**	24	40	4	**68**
**Retired physicians**	42	12	2	**56**
**TOTAL**	**138**	**182**	**302**	**622**

Among 484 physicians invited to participate by email, 302 responded (i.e., 62.4%). Among the 498 active prescribers belonging to the SFA, 296 participated in the survey (i.e., 59.4% of all active prescribers).

In total, 296 participants provided a response to the first and main question (‘do you prescribe baclofen for alcohol use disorders?’). Among the 296 participants for whom responses were analysed, 223 (75.3%) answered all of the questions of the survey, i.e., gave a response to each of the 2 questions that followed a ‘No’ answer to the first and main question or gave a response to each of the 19 questions that followed a ‘Yes’ answer to the first and main question.

The mean age of the participants was 53.4±8.6 years, and the study cohort was 42.5% female and 57.5% male. In total, 221 alcohol specialists (74.6%) declared that they prescribed baclofen in their clinical practice, and the remaining 75 (25.4%) answered that they did not (see **Table 1**). There was no significant difference between prescribers and non-prescribers in age (53.4±8.7 vs. 53.5±8.5 years; p = 0.85) or gender (43.4% vs. 40.6% females; p = 0.67). The raw response patterns to the survey are provided in **Table 1**.

**Figure 1:**
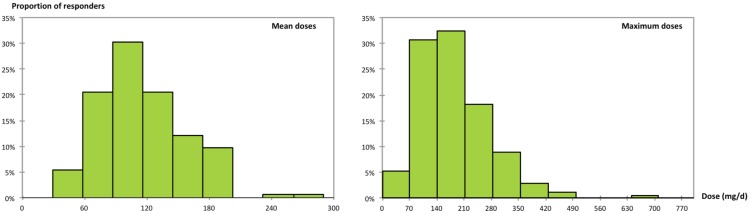
Repartitions of reported mean and maximum doses of baclofen used by prescribers of the survey. 71.5% of the reported mean doses were between 58 and 145 mg/d, while 81.2% of the reported maximum doses were between 71 and 284 mg/d. These findings highlight that baclofen was mainly used at high doses by French alcohol specialists.

The decile distribution of the mean and maximum prescribed doses among prescribers is shown in **Figure 1**.

There was no significant difference in mean age among the response clusters for question A (p = 0.34), which revealed that the number of patients treated with baclofen did not depend on the age of the physicians. However, the gender ratios were significantly different within the same clusters (p = 0.02); 47.1% of the female prescribers had prescribed baclofen to fewer than ten patients, and being male was associated with treating more patients with baclofen.

A direct comparison of the mean maximum dose between female and male prescribers revealed only a trend but no significant difference (170.95±77.7 mg/d vs. 199.71±100.3 mg/d; p = 0.07). However, the sex ratios were significantly different between the dose deciles in answer to question G (p = 0.03), and significantly more female prescribers were found in the lower maximum prescription dose answer categories.

Similarly, no significant difference in mean age was found among the different response clusters for question H (p = 0.85). Direct comparison of the mean baclofen doses prescribed by males and females revealed no difference (p = 0.18). The proportions of female prescribers were lower in the deciles of higher mean doses than in the deciles of lower mean doses (question H), but there was only a trend for a sex difference in the different dose-decile distributions concerning the answers to question H (p = 0.09).

Within the five ordered-category answers to question M, differences reflecting the number of patients treated by age, sex ratio, average mean and maximum doses prescribed, and category are shown in **Table 3**. There were significant correlations between the level of felt efficacy and the mean dose used (ρ = −0.35, p<10E-4) and between the level of felt efficacy and the maximum dose used (ρ = −0.42, p<10E-4). Last, the number of patients treated with HDB per physician (question A) and the level of felt efficacy (item M) were significantly correlated (ρ = −0.41, p<10E-4).

**Table 3:** Differences within ordered-category answer patterns for question M (“What is your personal opinion on baclofen’s efficacy, by comparison with approved treatments?”).

	1 = Much more efficacious	2 = More efficacious	3 = Equally efficacious	4 = Lessefficacious	5 = Much less efficacious	p-value
**Total number of responders (n)**	27	63	59	14	1	NA
**Percentage of total responders**	16.5%	38.4%	36%	8.5%	0.6%	NA
**Mean age of responders (years)**	54.3±7.8	52.9±8.2	51.9±7.8	48.7±10.2	56	0.28
**Percentage of women**	29.6%	42.9%	37.3%	26.7%	0%	0.52
**Averaged ordered-category matching the** **number of patients treated** (question A)	2.9±0.99	2±0.91	1.7±0.74	1.5±0.52	1	<10E-4
**Average mean dose used (mg/d)**	142.6±47.1	113±38.5	89.2±42.2	115.4±44.5	80	<10E-4
**Average maximum dose used (mg/d)**	286.3±125	193.7±74.4	166.4±88.7	173.6±72	120	<10E-4

Comparisons between the 5 ordered-category answers were made using Fisher exact tests for categorical variables and Kruskall-Wallis tests for quantitative variables. Differences in the response patterns to question A (ordered-categories defining the number of patients treated) within the 5 possible answers to question M were analysed by considering ordered-category answers to question A as a discrete continuous variable (from 1 to 4), and then using a Kruskall-Wallis test. By considering response patterns to both questions A and M as discrete continuous variables, we also calculated a Spearman’s correlation coefficient between these two variables (ρ = −0.41, p<10E-4), indicating that the more the prescribers treated patients with baclofen, the more they deemed that this drug was more efficacious than approved medications.

Concerning question N, i.e., the subjective feeling of the safety of HDB, there was a significant difference in the mean age of physicians within the different response clusters (p = 0.048). However, there was no statistical correlation within the entire sample between age and the subjective feeling of safety (ρ = −0.01, p = 0.24). There was also no correlation between the opinion of safety (item N) and the number of patients treated (item A) (ρ = 0, p = 0.93) nor between the opinion of safety (item N) and the dose escalation pattern (item E) (ρ = −0.1, p = 0.36). Last, no significant difference was found between the different response categories to question N regarding the gender ratio (p = 0.62), mean dose (p = 0.49), or maximum dose of treatment (p = 0.25).

## Discussion

While previous articles on HDB have essentially aimed at detailing the specific practices of diverse academic teams [9]–[11], [17], the present survey is the first to depict the HDB prescribing practices of a large number of clinicians. Such an investigation is important due to the aforementioned situation of both widespread and purely empirical use of sometimes extremely high doses of baclofen, despite the quasi-total lack of previously existing scientific evidence or regulation.

The first finding of the survey is that a large majority of participants did prescribe baclofen for AUDs. Moreover, they usually prescribed HDB, as 71.5% of the reported mean doses were between 58 and 145 mg/d, whilst 81.2% of the reported maximum doses were between 71 and 284 mg/d (see **Figure 1**). Baclofen was usually prescribed long term, as almost half of the prescribers used baclofen for at least 6 months (item I). However, most of the prescribers declared tapering doses over time (Item J). Such dose ranges and utilisation schemes were relatively similar to those found in previous cases or cohort studies published on HDB [7]–[11]. Despite the current lack of scientific evidence on both its efficacy and safety, only one-fourth of alcohol specialists preferred not to prescribe HDB, although primarily for this reason. This is an important result, as it highlights the diffusion of HDB prescribing practices among French alcohol specialists. Moreover, as shown by the responses to items C and D (see **Table 1**), HDB was equally used for promoting abstinence and for reducing alcohol consumption. This is congruent with previous surveys among SFA members, which found that reducing drinking was an actual care strategy, even in cases of alcohol dependence [19]. Moreover, these findings belie the statement occasionally given by some of the pro-baclofen associations in France, supposing that HDB is quasi-exclusively prescribed by private practice GPs, as French addiction specialists would be reluctant to do so, particularly for drinking reduction strategies. Nonetheless, the survey suggested that most of the participants did not use baclofen as a routine treatment; 79.2% of prescribers had treated less than 30 patients, and 67.8% did not use HDB as the first-line treatment. This suggests that the majority of participants used HDB sparingly and reserved it for a selected subgroup of their patients. Such an approach appeared more pronounced among female prescribers, who had significantly fewer patients on baclofen and used significantly lower mean and maximum doses. Interestingly, however, age was not found to be a factor determining baclofen prescription, and it also did not influence the doses used. This suggests that individual HDB prescribing practices are not generation dependent. Nonetheless, practices appeared very disparate regarding the doses and number of patients treated, suggesting that very individual factors may underlie such gaps in practice among prescribers.

The personal opinion of physicians regarding baclofen’s efficacy appeared significantly correlated with the number of patients treated and with the mean and maximum doses used (see **Table 3**). On the surface, this could be interpreted as a sign of an HDB dose-response effect. However, it could also mean the opposite, i.e., a consequence of physicians’ own beliefs, as prescribers who were the most convinced of baclofen’s efficacy might also be those who were more likely to treat the greatest number of patients and who used the highest doses. The forthcoming results of two on-going randomised controlled trials on HDB in France should help to precisely determine the meaning of such correlations. By contrast, individual opinions on HDB’s safety did not appear to be influenced by age, sex, or the intensity of the individual HDB prescribing practice. Nonetheless, 62.6% of prescribers considered HDB as less or much less safe than approved medications (see **Table 1**); on average, prescribers estimated that 29% of their patients had to discontinue baclofen because of the occurrence of ADRs. These HDB-related risks were also an important point for non-prescribers. Therefore, many participants in the survey, including HDB prescribers, reported some concerns regarding the management of the drug. This seems congruent with the increasing number of reports of previously known baclofen-induced ADRs as well as those of new ADRs that have been more recently described in the literature regarding the use of HDB in AUDs [20]–[24].

Despite the importance of such concerns, the survey found that the number of spontaneous reports of HDB-related ADRs to pharmacovigilance units or directly to the ANSM was dramatically low. Theoretically, the report of any serious ADRs is legally compulsory in France, but it is very likely that many physicians ignore the law on this point. This confirms a point raised in the 2012 national pharmacovigilance report on HDB [16], and more generally, it highlights the highly problematic nature of the lack of visibility regarding the safety aspects of off-label prescribing practices [25]. The fact that a majority of prescriptions did not include a mention of ‘hors-AMM’ is similarly troubling (question R; see **Table 1**). Unfortunately, the survey did not ask physicians whether they did not mention this intentionally, i.e., to ensure that patient can be reimbursed, or just because they were not aware that this mention was to be added. Such findings are important in the context of the forthcoming TRU measure, which will be a completely new procedure. Great effort in information diffusion and both patient and physician education will have to be made to succeed at framing HDB prescription and supervision. This should make patients and prescribers aware of their contributions to the correct feedback of safety data.

We acknowledge several limitations in this survey. First, the participants were all members of the SFA. In that sense, the present survey used a ‘chunk sampling’ strategy, which consists of defining a non-randomised sample in which participants are selected based on their accessibility [26]. Choosing other sampling methods was possible, particularly random sampling strategies, which may build the most representative samples, but only in cases with high response rates [26]. Here, such a strategy was particularly hard to conceive because there is no actual list including all French alcohol physicians, and even if such a list existed and if a random sample could have been invited to participate via mail or email, we assume that the response rate would have been much lower than that in the present survey. For this reason, we believe that the ‘chunk sampling’ method was the best compromise between representativeness and responsiveness. Nevertheless, we acknowledge that this choice could result in differences between the chosen sample and the French alcohol specialists who were not members of the SFA, regarding features such as age, gender, experience, type of medical practice, and vocational training.

Another limitation of the survey is that it investigated HDB prescribing practices and physicians’ personal opinions on the drug among only alcohol specialists. Though it may be hard to define what an ‘alcohol specialist’ is, a membership to the SFA as a clinician implies that a large part of the professional schedule is dedicated to meeting and treating patients with AUDs. SFA clinician members may thus be largely more informed on HDB than physicians with a more diversified clinical practice. Therefore, the response patterns could have been very different for private practice GPs, for whom addiction medicine does not constitute the primary activity but who might currently constitute the most important prescribers of HDB in France [27]. Consequently, the findings of the survey cannot be extrapolated to all physicians who prescribe HDB in France, as no similar survey has been conducted among French private practice GPs to date. Numerous additional questions would have been of great interest in the survey, including the management of other psychotropic drugs by physicians, the selection criteria for patients who may receive HDB prescriptions, and some elements concerning the opinions of physicians on the forthcoming TRU measure or on the coverage of baclofen in the French media. Such questions were finally excluded from the survey for feasibility reasons. Indeed, a compromise had to be found between the number of questions addressed and the amount of time that participants were ready to spend on the survey. Increasing the number of questions risked lowering the number of participants or responses.

The assessment of the overall quality of this survey is also restrained by the limited literature and the lack of consensus regarding the optimal drafting and reporting of survey research [28]. However, several criteria have been proposed for conducting good-quality surveys [26], [28]. The research design of the present survey met many of the required criteria, i.e., defining the main objective of the survey (prescription of baclofen), providing full access to questionnaire items (see **Table 1**), discussing the sample’s representativeness (see above), detailing the mode of sampling (chunk sampling), the mode of survey administration (online), the type and number of contact attempts (three consecutive emails), the methods for handling missing items, and the global response rate. Several other quality criteria were not fulfilled, including previous sample size calculation, the psychometric properties of the questionnaire, and the cut-off limit for partial completion of the questionnaire. Regarding the psychometric features of the questionnaire, a preliminary test could have been performed among baclofen prescribers and baclofen non-prescribers to determine whether the main question of the survey allowed adequately distinguishing prescribers from non-prescribers (validity criteria). Additionally, a test-retest procedure could have been envisaged to evaluate the reliability of the survey. However, the majority of published surveys do not provide these elements [28], which often require prior data. The overall response rate (62.4%), the response rate among invited prescribers (69.4%), and the ratio between the number of responders and the total number of prescribing physicians in the SFA (59.4%) all matched the usual quality standards among previously published surveys [26]. Overall, despite several methodological limitations, we believe that the presented survey met the majority of the requirements for evidence-based quality criteria.

In conclusion, the vast majority of the French alcohol specialists who participated in the survey prescribed baclofen to their patients, typically at off-label doses. However, the response patterns revealed that most of the physicians did not regularly prescribe HDB, as they limited the prescription of the drug to a small number of their patients. This could be related to the safety concerns that many of the participants reported with this drug or could be due to the off-label nature of this medication. Nonetheless, almost none of the prescribers had made a spontaneous report to the health security system, although reporting serious events is compulsory. The forthcoming TRU measure for framing HDB off-label prescriptions in France should be the initiation of a sustained effort to educate both patients and prescribers on the pharmacovigilance system. Moreover, off-label HDB prescribing practices may spread beyond France in the future, as they have already been reported in other countries, notably adjacent European countries such as Belgium or Switzerland [29], [30], as well as in Australia [9] and the USA [8]. In that sense, France should serve as an example to depict, in future investigations, the dissemination of off-label prescribing practices among physicians and to better understand what drives physicians to use off-label medications at uninvestigated doses and why they sometimes may not feel the need to monitor or report safety concerns. Such issues go far beyond the mere question of HDB, as they may address all types of off-label prescribing practices and should interest regulatory authorities and decision makers regarding public health strategies and medical education.
